# Neutralization of Streptolysin S-Dependent and Independent Inflammatory Cytokine IL-1β Activity Reduces Pathology During Early Group A *Streptococcal* Skin Infection

**DOI:** 10.3389/fcimb.2018.00211

**Published:** 2018-07-03

**Authors:** Rebecca A. Flaherty, Deborah L. Donahue, Katelyn E. Carothers, Jessica N. Ross, Victoria A. Ploplis, Francis J. Castellino, Shaun W. Lee

**Affiliations:** ^1^Department of Biological Sciences, University of Notre Dame, Notre Dame, IN, United States; ^2^Eck Institute for Global Health, University of Notre Dame, Notre Dame, IN, United States; ^3^W. M. Keck Center for Transgene Research, University of Notre Dame, Notre Dame, IN, United States

**Keywords:** Streptolysin S, host-pathogen interactions, inflammatory signaling, p38 MAPK, cytokine, interleukin-1 beta, subcutaneous infection

## Abstract

The bacterial pathogen Group A *Streptococcus* (GAS) has been shown to induce a variety of human diseases ranging in severity from pharyngitis to toxic shock syndrome and necrotizing fasciitis. GAS produces a powerful peptide toxin known as Streptolysin S (SLS). Though long recognized as a potent cytolysin, recent evidence from our lab has shown that SLS-dependent cytotoxicity is mediated through activation of the pro-inflammatory mediators p38 MAPK and NFκB. These findings led us to hypothesize that activation of p38 MAPK and NFκB signaling drive the production of pro-inflammatory cytokines which, in turn, serve as positive feedback signals to initiate cytotoxicity in infected host cells. To address this hypothesis, we utilized a cytokine array to characterize the SLS-dependent pro-inflammatory cytokine response to GAS infection in human keratinocytes. From these studies, IL-1β was found to be markedly upregulated in the presence of SLS, and further investigation revealed that this cytokine contributes to cytotoxicity in human keratinocytes during infection. Subcutaneous infection studies were performed in mice to address the physiological impact of increased IL-1β production. These studies demonstrated that IL-1β is produced during GAS skin infection in an SLS-dependent manner. Furthermore, inhibition of this cytokine and the upstream kinases and other signaling mediators that drive its production reduced SLS-mediated lesion formation early in the infection process. Together, our findings indicate that pharmacological inhibition of this inflammatory axis holds promise as a therapeutic strategy to reduce tissue destruction during severe invasive Group A *Streptococcal* infections.

## Author summary

Group A *Streptococcus* (GAS) is one of the most significant global pathogens, causing many common symptoms such as pharyngitis, cellulitis, and impetigo. It is also responsible for more severe diseases such as rheumatic fever, necrotizing fasciitis, and toxic shock syndrome. A landmark feature of GAS is its ability to produce a powerful toxin known as Streptolysin S (SLS). SLS is a small, cytotoxic peptide whose pronounced ability to rapidly lyse red blood cells was first observed over a century ago. It has been widely held that SLS acts as a major contributing factor during invasive Group A *Streptococcus* infection through rapid destruction of cells and tissues during the infection process. However, studies by our laboratory and others suggest that SLS may also play a more complex role in disease at physiologically relevant levels, including its ability to precisely target and inactivate host proteins. In this study, we characterize the SLS-dependent pro-inflammatory cytokine response to GAS infection in human keratinocytes. Our results show that activation of the specific cytokine, IL-1β, was found to be a significant contributor to GAS pathogenesis. Inhibiting the activity of IL-1β during infection reduced SLS-mediated lesion pathology in an animal model. Our findings provide key insights into the role of SLS in GAS disease, and may provide a novel therapeutic avenue to treating severe invasive GAS infections.

## Introduction

The bacterial pathogen Group A *Streptococcus* (GAS) has been shown to induce a variety of human diseases including mild conditions of the skin and mucosal membranes, such as impetigo and pharyngitis, as well as life-threatening infections, such as toxic shock syndrome and necrotizing fasciitis (Cunningham, 2000; Carapetis et al., 2005a,b; Walker et al., 2014). One of the major virulence factors produced by GAS is the peptide toxin Streptolysin S (SLS) (Datta et al., 2005; Lee et al., 2008; Mitchell et al., 2009; Molloy et al., 2011). Though this toxin has long been recognized as a potent cytolysin (Todd, 1938; Bernheimer and Schwartz, 1964; Keiser et al., 1964; Ofek et al., 1972; Hryniewicz and Pryjma, 1977; Carr et al., 2001; Sierig et al., 2003; Datta et al., 2005; Miyoshi-Akiyama et al., 2005; Goldmann et al., 2009), recent evidence from our laboratory has indicated that SLS-dependent cytotoxicity in keratinocytes is mediated through inactivation of the cytoprotective factor Akt1 and subsequent activation of p38 MAPK and NFκB (Flaherty et al., 2015). These signaling events ultimately lead to cell death through a form of programmed necrosis, which is dependent on RIPK1 and RIPK3 (Flaherty et al., 2015). From these findings, we hypothesized that activation of p38 MAPK and NFκB signaling drive the production of pro-inflammatory cytokines which, in turn, serve as positive feedback signals to initiate programmed necrosis in infected host cells (Flaherty et al., 2015). The goal of the current studies described here was to characterize SLS-mediated cytokine production during GAS skin infection to better understand the link between toxin-mediated inflammatory responses and subsequent cell death and tissue destruction.

IL-1β is a potent inflammatory cytokine that is released from host cells when cellular damage occurs and aids in the regulation of a variety of immunological responses including prostaglandin synthesis, cytokine production, neutrophil influx and activation, T-cell and B-cell activation, fibroblast proliferation, collagen production, and the differentiation of Th17 T-cells (Dinarello, 1996; Sahoo et al., 2011; Dinarello et al., 2012; LaRock and Nizet, 2015). The canonical pathway for IL-1β production is initiated by inflammasome activation (Kapur et al., 1993; Dinarello, 1996, 2009; Valderrama et al., 2017). IL-1β is produced in a pro-form, which requires cleavage to a mature form by caspase-1 (Kapur et al., 1993; Dinarello, 1996, 2009; Valderrama et al., 2017). Of note, alternative enzymes such as SpeB, produced by GAS, have been shown to be capable of cleaving pro-IL-1 β to its mature form independently of caspase-1 (Kapur et al., 1993; LaRock et al., 2016). This cytokine is critical for inducing fever and the acute-phase responses following a local inflammatory event, and it has been shown to provide a protective effect for the host in response to a variety of bacterial, viral and fungal infections (Dinarello, 1996, 2009; Dinarello and Fantuzzi, 2003; Sahoo et al., 2011; Dinarello et al., 2012; LaRock and Nizet, 2015). However, overproduction of IL-1β can produce highly deleterious effects in the host. IL-1β has been shown to play an important role in driving the pathogenesis of numerous microbial infections, such *Escherichia coli*-induced sepsis, severe lung inflammation during influenza infection, *Pseudomonas aeruginosa-*induced pneumonia, *Burkholderia pseudomallei*-induced melioidosis, and *Porphyromonas gingivalis-*mediated periodontal tissue destruction (Schultz et al., 2002, 2003; Sahoo et al., 2011; Kato et al., 2014; Vanden Berghe et al., 2014; Kim et al., 2015). In a mouse model of necrotizing soft tissue infection (NSTI) produced by GAS, Chella Krishnan et al. utilized a forward systems genetics to determine host gene expression profiles relevant to the NSTI, and revealed IL-1β production to be significantly associated with NSTI pathogenesis by GAS (Chella Krishnan et al., 2016). Patient studies have also revealed IL-1β to be a major driver associated with severe GAS infections such as NSTI, especially with 30-day mortality (Chella Krishnan et al., 2016; Hansen et al., 2017). Several GAS virulence factors, including M protein, SpeB, SpeA, and SLO have been previously identified as important contributors to IL-1β production during severe GAS infection (Hackett and Stevens, 1992; LaRock et al., 2016; Valderrama et al., 2017). SpeA and SLO have been shown to trigger potent interleukin-1 production in human monocyte cell lines (Hackett and Stevens, 1992). Recently, Valderama et al. identified soluble forms of M1 protein as a specific trigger for caspase-1-dependent NLRP3 inflammasome activation, which resulted in the maturation and release of IL-1β (Valderrama et al., 2017). These studies suggest that IL-1β plays a central role in GAS pathogenesis; however, host-context interactions, GAS infection model systems, and strain specific isoforms, will likely further define the exact role of IL-1β production in disease. Finally, IL-1β production is known to be upregulated in a number of autoimmune inflammatory disorders, such as osteoarthritis, rheumatoid arthritis, gout, psoriasis, and type II diabetes (Dinarello, 1996; Dinarello et al., 2012; Horneff, 2013). Genetic knock-out mouse models and IL-1β neutralizing therapies have been shown to alleviate the damaging effects of this cytokine both in response to microbial infections and in autoimmune-mediated disease states (Dinarello, 1996; Sahoo et al., 2011; Dinarello et al., 2012; Horneff, 2013; Vanden Berghe et al., 2014; Kim et al., 2015; Spohn et al., 2016).

To elucidate the effects of SLS-mediated inflammatory signaling in epithelial keratinocytes, we performed cytokine array studies to identify inflammatory cytokines that are produced by keratinocytes in response to GAS infection. These studies revealed several host cytokines that are differentially regulated in response to GAS infection compared to uninfected cells, as well as several cytokines whose production is altered in an SLS-dependent manner. The most notable SLS-mediated alteration in keratinocyte cytokine production observed was a significant increase in the production of the key pro-inflammatory cytokine interleukin 1-beta (IL-1β). Studies using p38 MAPK and NFκB inhibitors demonstrated that IL-1β is activated downstream of these pro-inflammatory signaling mediators.

To further address the physiological impact of increased IL-1β production during GAS infection, we performed *in vitro* studies in human keratinocytes as well as *in vivo* assessments in a humanized mouse model of GAS subcutaneous infection. These studies revealed that the SLS-mediated increase in IL-1β production leads to increased cytotoxicity *in vitro*, which can be significantly reduced through the use of IL-1β-specific neutralizing antibodies. Data from our *in vivo* studies demonstrated that IL-1β is robustly induced during GAS skin infection in both an SLS-dependent and independent manner, and that inhibition of this inflammatory cascade leads to reduced skin lesion formation early in the infection process. These data indicate that pharmacological targeting of IL-1β and its upstream mediators may be a useful strategy to reduce tissue damage during invasive GAS infections.

## Materials and methods

### Bacterial cultures

The GAS M1T1 5448 strains utilized for these experiments included WT GAS, a *sagA*Δ*cat* SLS-deficient mutant (Δ*sagA*), and a *sagA* complemented strain. The *sagA*Δ*cat* GAS strain was generated by the lab of Victor Nizet at the University of California, San Diego (Datta et al., 2005), and the complemented strain was generated in our lab by transformation of Δ*sagA* with the pDCerm expression vector containing the wild-type *sagA* gene along with 300 bp directly upstream of *sagA* to include native promoter sequences (abbreviated in text as Δ*sagA*+*sagA*) (Higashi et al., 2016). GAS M1T1 5448 strains were grown in Todd-Hewitt broth at 37°C for 16–20 h prior to infecting human cells or injecting into mice.

### Epithelial cell culture

HaCaT human epithelial keratinocytes (Boukamp et al., 1988), a kind gift from V. Nizet, were used for all studies involving immortalized cell lines. Cells were maintained in Dulbecco's Modified Eagle's Medium (DMEM) (Life Technologies 11995-073) with 10% heat inactivated fetal bovine serum (FBS) and incubated at 37°C with 5% CO_2_. HaCaT cells were maintained in 100 mm culture dishes (Nunc).

### Keratinocyte infection

HaCaT cells were grown to 90% confluency in 6 or 24 well tissue culture plates (CytoOne) or in 100 mm dishes (Nunc). Immediately prior to treatment, the cells were washed with sterile PBS and fresh media was applied (DMEM + 10%FBS). Overnight bacterial cultures of GAS were centrifuged and resuspended in fresh Todd-Hewitt broth and their optical densities (OD_600nm_) were normalized. The HaCaT cells were infected with the normalized overnight cultures of wild-type, Δ*sagA* or *sagA* complemented (Δ*sagA* + *sagA*) GAS at a MOI of 10 bacteria per host cell. The infected cells were incubated at 37°C with 5% CO_2_ for times indicated in each experiment.

### Ethidium homodimer cell death assay

HaCaT cells were plated in 24 well tissue culture plates and infected using the conditions described above (MOI = 10). After infection, the cells were washed with sterile PBS. The cells were then covered and incubated at room temperature for 30 min with 4 μM Ethidium Homodimer 1 (Molecular Probes) in PBS. The level of fluorescence was determined using a plate reader set to 528 nm excitation and 617 nm emission with a cut-off value of 590 nm. The percentage of dead cells was determined by adding 0.1% (w/v) Saponin (Sigma) to each well following the initial reading and allowing the plate to incubate for an additional 20 min at room temperature before reading the plate a second time at the same settings. Percent membrane permeabilization values were obtained individually for each well by dividing the initial fluorescence reading (post-treatment) by the second fluorescence reading (post-Saponin). Each treatment condition was performed in triplicate (at minimum), and the average and standard deviation of each condition were plotted for comparison. Normal distribution was assumed, and significance was determined by ANOVA, followed by post-ANOVA Dunnett's tests to compare each condition to a corresponding control mean as indicated.

### Inhibitor compounds and neutralizing antibodies

SB203580, which inhibits p38 MAPK activity, was obtained from Cell Signaling. The NFκB inhibitor, curcumin, was obtained from Santa Cruz Biotechnology. Interleukin-1 beta neutralizing antibodies (specific for mature mouse or mature human IL-1 beta) and the rabbit polyclonal isotype control antibodies were obtained from Abcam.

### Statistical analyses

All statistical analyses were carried out using Graph Pad Prism 6.0. Significant differences for individual pairs of means were determined by Student's *t*-test, and *p*-values of < 0.05 were considered to be significant. For data sets in which means of 3 or more groups were being compared, ANOVA testing was used to determine overall *p*-values. In cases where a significant *p*-value (*p* < 0.05) was obtained by ANOVA, *post hoc* Dunnett's tests were carried out to compare the means of all members of a data set to a control mean, which is indicated for each experiment. *P*-values of < 0.05 were considered to be significant. Individual *p*-values from *t*-tests and *post hoc* Dunnett's tests were reported as follows for all data sets ^*^, *p* = 0.01–0.05; ^**^, *p* = 0.001–0.01; ^***^, *p* = 0.0001–0.001; ^****^, *p* < 0.0001. For the *in vivo* analyses, values that were greater than or less than 2 times the standard deviation of the mean for the complete data set were determined to be outliers and were therefore excluded.

### Cytokine array

HaCaT keratinocytes were infected with wild-type or SLS-mutant GAS via the direct infection protocol described above at MOI of 10. The cell culture media was collected 6 h post-infection. The cell culture media was immediately centrifuged (2,400 rcf for 10 min) to remove the majority of the bacteria from the samples, and the supernatants were collected into fresh tubes and stored at −20°C until sample analysis. Immediately prior to sample analysis, samples were thawed on ice and centrifuged at 16,000 rcf for 5 min to remove any remaining cellular or bacterial debris. The Abcam Human Cytokine Antibody Array Kit (ab133998), which allows for the detection 80 human cytokines, was utilized to determine relative cytokine levels in the cell culture samples. All steps of the sample analysis procedure were performed according to the manufacturer's instructions. Following detection of cytokine protein levels on film, densitometry was performed to determine relative protein levels of each cytokine. These experiments were performed in two phases. The first experiment compared cytokine levels from keratinocytes infected with WT GAS to mock-infected keratinocytes, and the second experiment compared cytokine levels between keratinocytes infected with SLS-deficient GAS to SLS-complemented GAS infection.

### Mouse skin infections

Human plasminogen expressing male mice, C57B1/6(hPg(Tg)), at 6–8 weeks of age, were utilized for each of the studies shown here. For studies involving inhibition of IL-1β production or activity, mice were treated as follows. Mice being treated with the p38 MAPK inhibitor, SB203580, or the NFκB inhibitor, curcumin, received 50 mg/kg of the respective compound in 9% DMSO, 9% Tween 80, and 0.7% saline via intraperitoneal injection (220 μL injection volume). Mice being treated with IL-1β neutralizing antibody (ab9722) or pAb isotype control antibody received 100 μg (220 μL injection volume in 0.7% saline) of the antibody. Vehicle controls (9% DMSO, 9% Tween 80, and 0.7% saline) were also included, and the treatment regimens were performed in a blinded setting. The treatments were administered only once, and were co-injected subcutaneously along with the bacteria at the time the infection was initiated.

To initiate necrotizing skin infection, mice were injected subcutaneously in the flank with 10^7^ CFUs of M1T1 5448 GAS. Bacteria used in these experiments were subcultured from overnight cultures and grown to log phase (OD_600_ = 0.5) in Todd-Hewitt broth. Prior to injection, the cultures were washed, resuspended in sterile 0.7% saline, and normalized to the same optical density to achieve the desired CFU for infection. Mice were observed for 24–72 hr, and the formation of necrotic ulcers was monitored daily. Production of IL-1β both in mouse skin tissue and in mouse plasma was determined at the time points indicated, and sample processing and analysis is described below.

### Cytokine detection by ELISA

#### Human keratinocytes

HaCaT keratinocytes were infected with wild-type or SLS-mutant GAS (Δ*sagA*) via the infection protocol described above at MOI of 10. The cell culture media was collected 6 h post-infection. The cell culture media was immediately centrifuged (2,400 rcf for 10 min) to remove the majority of the bacteria from the samples, and the supernatants were collected into fresh tubes and stored at −20°C or used immediately. Prior to sample analysis, samples were thawed on ice and centrifuged at 16,000 rcf for 5 min to remove any remaining cellular or bacterial debris. For conditions in which neutralizing antibodies were utilized, samples were incubated overnight at 4°C with protein A/G beads (Pierce) to capture IL-1β/antibody complexes. These samples were centrifuged at 200 rcf for 5 min to remove the beads and bound protein complexes prior to loading onto the ELISA plate. The Abcam IL-1 beta Human ELISA Kit (ab100562) was utilized to quantify the amount of IL-1β produced by HaCaT cells following infection with GAS. To our knowledge, this kit does not discriminate between the production of pro-IL-1β and mature IL-1β. The Abcam TIMP-2 Human ELISA Kit (ab188395) was utilized to quantify TIMP-2 under the same infection conditions. All steps of the sample analysis procedures were performed according to the manufacturer's instructions. Data from three independent biological replicates, with four technical replicates per sample, were pooled to determine the average IL-1β produced following each of the infection and treatment conditions shown. Data from three independent biological replicates, with three technical replicates per sample, were pooled to determine the average TIMP-2 produced for each infection and treatment condition shown. The average and standard deviation of each condition were plotted for comparison. Normal distribution was assumed, and significance was determined by ANOVA, followed by post-ANOVA Dunnett's tests to compare each condition to a corresponding control mean as indicated.

#### Mouse tissues

Following skin infection, tissue from the infection site was collected and immediately stored at −80°C. At the time of analysis, the tissue samples were thawed on ice and homogenized in 1 mL of lysis buffer (provided in Abcam ab197742 Mouse IL-1β ELISA Kit) to solubilize IL-1β. The protein concentration of each homogenized skin sample was determined by BCA assay (Pierce) using BSA protein standards and normalized prior to loading the samples onto a 96-well ELISA plate specific for IL-1β detection. The Abcam Mouse IL-1 beta ELISA SimpleStep Kit (ab197742) was utilized to quantify the amount of IL-1β present in the mouse skin tissue following GAS infection. To our knowledge, this kit does not discriminate between the production of pro-IL-1β and mature IL-1β. All steps of the sample analysis procedure were performed according to the manufacturer's instructions. The average and standard deviation of each condition (*n* = 4 mice) were plotted for comparison. Normal distribution was assumed, and significance was determined by ANOVA, followed by post-ANOVA Dunnett's tests to compare each condition to a corresponding control mean as indicated.

#### Mouse plasma

Following skin infection, plasma samples from each mouse were collected, citrated, and immediately stored at −20°C. At the time of analysis, the samples were thawed on ice and centrifuged at 2,000 rcf for 10 min. The Abcam Mouse IL-1 beta ELISA SimpleStep Kit (ab197742) was utilized to quantify the amount of IL-1β present in the mouse plasma following GAS infection. All steps of the sample analysis were performed according to the manufacturer's instructions. The average and standard deviation of each condition (*n* = 4 mice) were plotted for comparison. Normal distribution was assumed, and significance was determined by ANOVA, followed by post-ANOVA Dunnett's tests to compare each condition to a corresponding control mean as indicated.

### Determining colony forming units (CFUs) at the infection site

Following infection, tissue samples from each mouse (*n* = 6) were collected and immediately homogenized in 1 mL of 0.7% sterile saline. Samples from each mouse were serially diluted in sterile saline and plated in quadruplicate on Todd-Hewitt agar. The bacteria were grown at 37°C overnight and the resultant colonies were counted to determine the CFUs at the wound site for each infection condition.

### Ethics statement

Animal protocols were approved by the Institutional Animal Care and Use Committee (IACUC) of the University of Notre Dame (IACUC approved protocol number 16-02-2965, expires 4/20/2019). All animal facilities are AAALAC accredited (PHS Animal Welfare Assurance A3093-01).

## Results

### Group A *Streptococcus* influences keratinocyte production of inflammatory cytokines during infection

**Figure 1:**
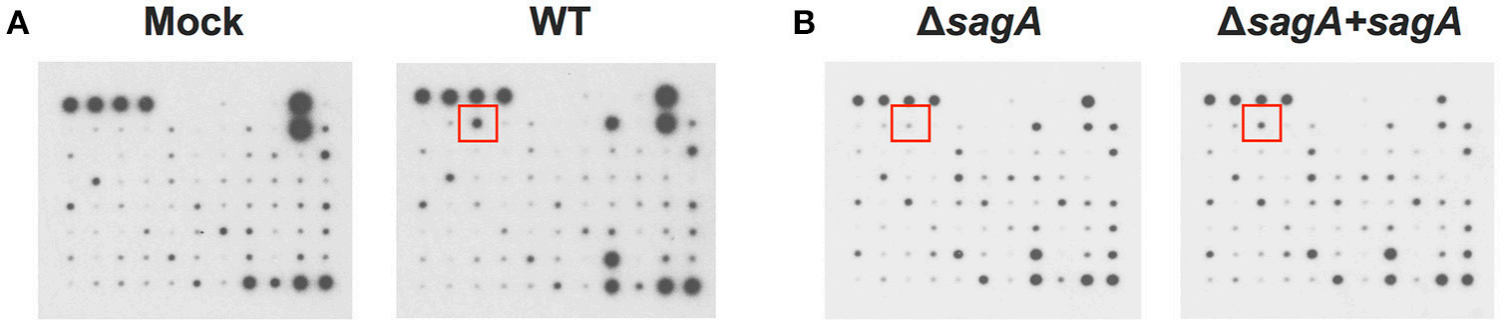
Group A *Streptococcal* infection modulates keratinocyte inflammatory cytokine production. HaCaT human keratinocytes were either mock infected or infected with GAS at an MOI of 10 for 6 h, and cell culture media was collected for cytokine analysis. **(A)** Cytokine production from uninfected cells was compared with WT-infected keratinocytes to identify GAS-dependent changes in cytokine signaling. **(B)** Cytokine production from SLS-deficient GAS infection was then compared to that induced by *sagA* complemented infection to specifically identify SLS-dependent changes. Red squares denote location of IL-1β cytokine signal position in the array.

To elucidate the effects of SLS-mediated inflammatory signaling in epithelial keratinocytes (HaCaT cells), we performed cytokine array studies to identify inflammatory cytokines that are produced by keratinocytes in response to GAS infection. Based on our previous work, we hypothesized that SLS-mediated cytotoxicity in keratinocytes may be induced following the production of pro-inflammatory cytokines in infected host cells (Flaherty et al., 2015). As programmed cell death in GAS-infected keratinocytes was readily detectable by 6 h post-infection at an MOI of 10, we began our analysis by collecting the cell culture media produced by keratinocytes infected with wild-type GAS or mock infected under these same infection conditions (Figure 1A) (Flaherty et al., 2015). Densitometry was performed on the two arrays, and these values were used to compare the relative cytokine levels induced in keratinocytes during WT or mock infection (Table 1). In cases where the densitometry values of both numbers being compared were below the average negative control value, the fold change was set to 1.0 to minimize confounding results. Results from these analyses indicated that GAS induces a 2-fold or greater increase in GM-CSF, IL-1β, IL-6, and MIF in WT vs. mock infection (Table 1, Table S1). Additionally, GAS induces a 2-fold or greater loss in MDC, MIP-1δ, Osteoprotegerin, and TIMP-2 compared to mock-infected cells (Table 1).

**Table 1:** Fold changes in cytokine levels following Group A *Streptococcal* infection of keratinocytes.

**ROW 1**	**POS**	**POS**	**POS**	**POS**	**NEG**	**NEG**	**ENA-78**	**GCSF**	**GM-CSF**	**GRO**	**GRO-α**
WT/Mock	0.93	0.92	1.03	1.01	1.00	1.00	1.04	1.00	2.62	0.91	1.32
ΔsagA+sagA/ΔsagA	1.17	1.20	1.19	1.27	1.00	1.00	1.23	0.78	0.66	0.64	1.66
**ROW 2**	**I-309**	**IL-1**α	**IL-1**β	**IL-2**	**IL-3**	**IL-4**	**IL-5**	**IL-6**	**IL-7**	**IL-8**	**IL-10**
WT/Mock	1.09	1.10	4.57	0.92	0.64	0.97	1.84	6.92	1.42	0.81	1.14
ΔsagA+sagA/ΔsagA	2.32	2.03	2.98	2.35	1.76	1.49	1.22	0.71	1.06	0.98	1.27
**ROW 3**	**IL-12**	**IL-13**	**IL-15**	**IFN-**γ	**MCP-1**	**MCP-2**	**MCP-3**	**MCSF**	**MDC**	**MIG**	**MIP-1b**
WT/Mock	1.01	1.32	1.86	1.06	0.78	1.03	1.15	1.05	0.47	0.73	1.11
ΔsagA+sagA/ΔsagA	1.33	1.70	2.37	0.96	0.30	1.10	5.25	0.27	0.49	2.92	0.34
**ROW 4**	**MIP-1**δ	**RANTES**	**SCF**	**SDF-1**	**TARC**	**TGF-**β**1**	**TNF-**α	**TNF-**β	**EGF**	**IGF-I**	**Angio-genin**
WT/Mock	0.44	1.03	1.39	0.54	0.66	1.24	1.06	0.74	0.92	0.61	0.67
ΔsagA+sagA/ΔsagA	3.38	1.83	0.87	0.86	0.97	0.69	1.12	1.38	1.79	1.55	1.27
**ROW 5**	**Oncostatin M**	**Thrombo-poietin**	**VEGF**	**PDGF-BB**	**Leptin**	**BNDF**	**BLC**	**Ck** β **8-1**	**Eotaxin**	**Eotaxin 2**	**Eotaxin 3**
WT/Mock	0.95	0.55	1.29	0.22	1.07	0.83	0.89	0.72	1.73	1.28	1.11
ΔsagA+sagA/ΔsagA	0.98	0.43	1.05	1.67	0.87	0.89	1.17	0.96	1.13	1.04	0.87
**ROW 6**	**FGF-4**	**FGF-6**	**FGF-7**	**FGF-9**	**Flt-3 Ligand**	**Fractal-kine**	**GCP-2**	**GDNF**	**HGF**	**IGFBP-1**	**IGFBP-2**
WT/Mock	1.14	0.93	0.77	0.92	1.43	0.64	0.73	1.30	0.83	1.35	1.07
ΔsagA+sagA/ΔsagA	0.83	0.94	0.97	1.01	1.43	1.01	1.00	0.89	0.90	0.93	0.94
**ROW 7**	**IGFBP-3**	**IGFBP-4**	**IL-16**	**IP-10**	**LIF**	**LIGHT**	**MCP-4**	**MIF**	**MIP-3**α	**NAP-2**	**NT-3**
WT/Mock	0.90	1.37	1.05	0.85	1.22	1.22	0.76	5.52	1.30	1.36	1.71
ΔsagA+sagA/ΔsagA	1.25	1.44	1.21	1.04	1.22	1.04	1.00	1.06	1.00	1.25	1.10
**ROW 8**	**NT-4**	**Osteo-pontin**	**Osteo-protegerin**	**PARC**	**PIGF**	**TGF-**β**2**	**TGF-**β**3**	**TIMP-1**	**TIMP-2**	**POS**	**POS**
WT/Mock	1.34	0.55	0.44	0.71	0.96	0.97	0.82	1.00	0.41	0.91	0.98
ΔsagA+sagA/ΔsagA	1.51	0.84	0.66	0.72	1.76	1.18	1.26	0.85	0.41	0.91	0.92

*The red values are significantly upregulated, and the blue values are significantly downregulated*.

To determine which of these changes might be dependent on SLS production, we performed a second array under the same conditions to compare the keratinocyte cytokine response to infection with Δ*sagA* or *sagA* complemented GAS (Figure 1B). As with the WT vs. mock infection, densitometry was performed to allow for comparison of cytokine levels induced in keratinocytes following Δ*sagA* or *sagA* complemented infection (Table 1, Table S1). Results from these analyses indicated that *sagA* complemented GAS induces a 2-fold or greater increase in I-309, IL-1α, IL-1β, IL-2, IL-15, MCP-3, MIG, and MIP-1δ compared to the SLS-deficient mutant infection. Additionally, *sagA* complemented GAS induces a 2-fold or greater loss in MCP-1, MCSF, MDC, MIP-1b, Thrombopoietin, and TIMP-2 compared to the SLS-deficient mutant infection (Table 1, Table S1). In comparing the data from the two array experiments, we noted that the only cytokine found to be upregulated by 2-fold or more in both wild-type and *sagA* complemented infection compared to mock and SLS-deficient infection, respectively, was IL-1β (Table 1, Table S1). Additionally, the only two cytokines found to be reduced by 2-fold or more in both wild-type and *sagA* complemented infection compared to mock and SLS-deficient infection, respectively, were MDC and TIMP-2 (Table 1). Interestingly, TIMP-2 has been previously shown to be regulated by IL-1 β in a variety of cell types (Xue et al., 2003; Robert et al., 2016). The conserved trend in the production of these cytokines between the two array sets provides reasonable evidence that the observed effects in their production are SLS dependent. As IL-1β has previously been identified as an important cytokine contributing to the pathology of numerous disease states of GAS infections, we focused our subsequent studies on evaluating its role in the pathogenesis of GAS infection of the skin (Hackett and Stevens, 1992; Dinarello, 1996, 2009; Schultz et al., 2002; Dinarello and Fantuzzi, 2003; Sahoo et al., 2011; Dinarello et al., 2012; Horneff, 2013; Vanden Berghe et al., 2014; Kim et al., 2015; LaRock and Nizet, 2015; LaRock et al., 2016; Valderrama et al., 2017).

### Streptolysin S drives IL-1β production in human keratinocytes

**Figure 2:**
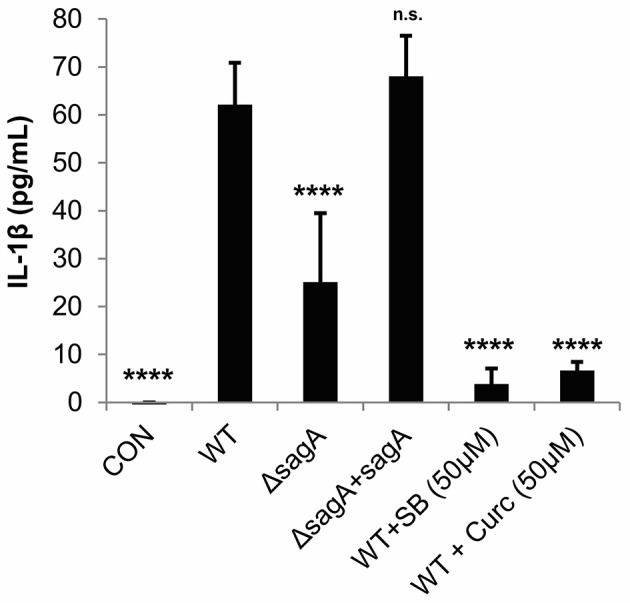
Streptolysin S induces IL-1β production in epithelial keratinocytes downstream of the p38/NFκB signaling pathway. HaCaT human keratinocytes were infected with GAS at an MOI of 10 for 6 h, and cell culture media was collected for cytokine analysis. Treatments to inhibit p38 MAPK (SB203580) or NFκB (curcumin) were applied 1.5 h prior to wild-type infection. The average of 3 biological replicates, each with 4 technical replicates, are represented for each condition, with error bars representing standard deviation. The overall *p*-value was determined by ANOVA (*p* < 0.0001). Dunnett's tests were performed to compare each condition to wild-type infection.

To verify that IL-1β is produced by GAS-infected keratinocytes in an SLS-dependent manner, we infected HaCaT cells with WT, SLS-deficient (Δ*sagA*) or *sagA* complemented (Δ*sagA* + *sagA*) bacteria and collected the cell culture media 6 h post-infection. An ELISA was performed to quantify the amount of IL-1β being produced under each infection condition. Robust production of this cytokine by SLS-producing strains of GAS was observed, with significantly reduced IL-1β production in the presence of the Δ*sagA* mutant or following mock infection (Figure 2). Based on our previous studies, we hypothesized that IL-1β production during GAS infection was occurring downstream of the p38 MAPK/NFκB pathway (Flaherty et al., 2015). As treatments with the p38 inhibitor, SB203580, and the NFκB inhibitor, curcumin, prior to infection were able to almost completely abrogate IL-1β production during GAS infection, we concluded that IL-1β is, indeed, produced in response to activation of this inflammatory pathway (Figure 2).

**Figure 3:**
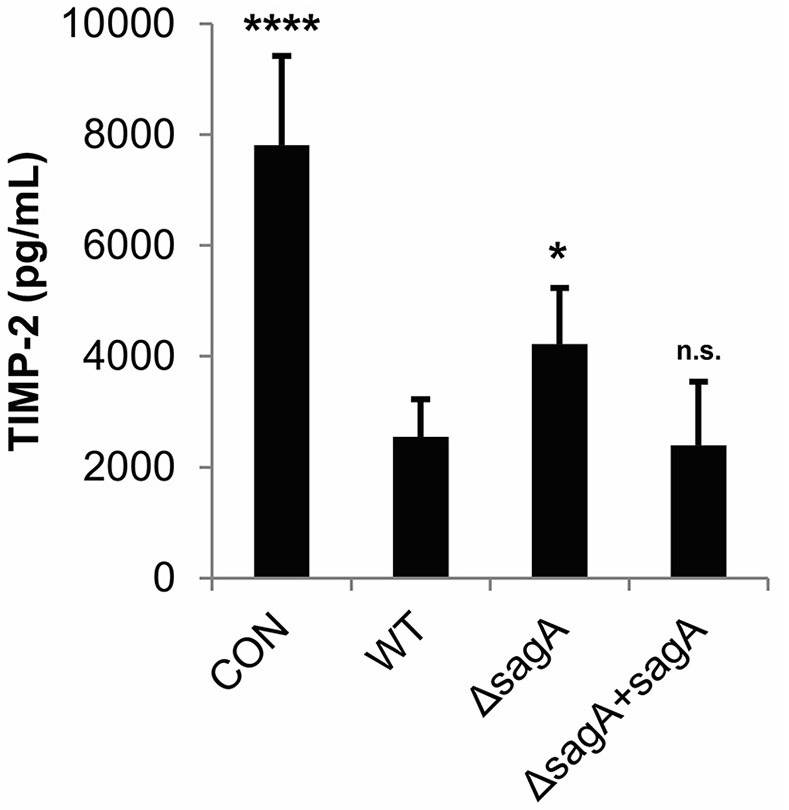
Streptolysin S contributes to loss of TIMP-2 during GAS infection. HaCaT human keratinocytes were infected with GAS at an MOI of 10 for 6 h, and cell culture media was collected for cytokine analysis. The average of 3 biological replicates, each with 3 technical replicates, are represented for each condition, with error bars representing standard deviation. The overall *p*-value was determined by ANOVA (*p* < 0.0001). Dunnett's tests were performed to compare each condition to wild-type infection.

TIMP-2, which was found to be significantly reduced in our cytokine array analysis in response to SLS, has been shown to be negatively regulated by IL-1β in response to various stimuli (Xue et al., 2003; Robert et al., 2016). We used an ELISA to confirm SLS-dependent loss of TIMP-2 during GAS infection (Figure 3). As TIMP-2 plays a role in the regulation of MMPs, we hypothesize that TIMP-2 loss may contribute to the tissue destruction that occurs during severe infection.

### IL-1β contributes to keratinocyte cytotoxicity during GAS infection

**Figure 4:**
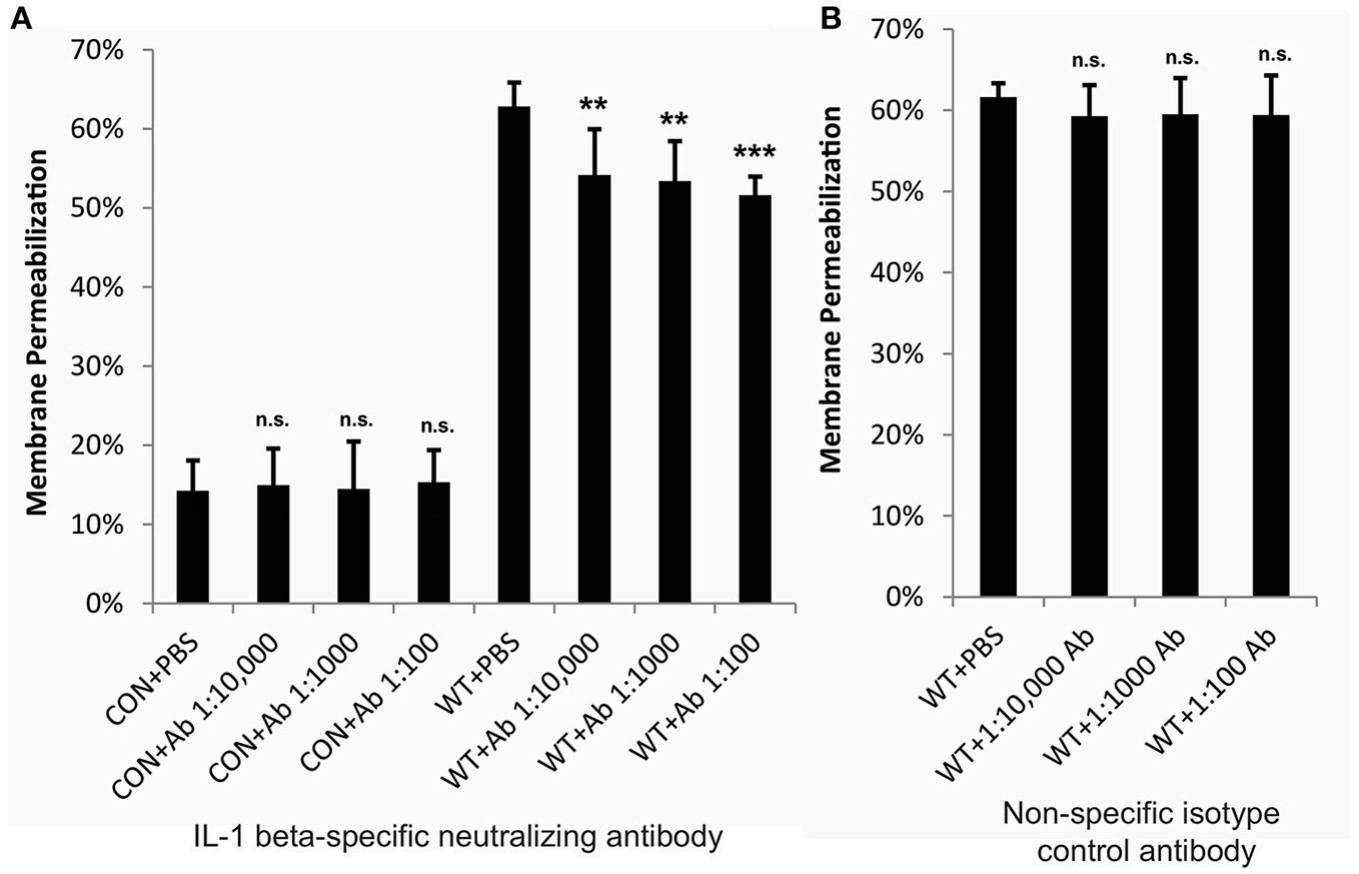
IL-1β contributes to cytotoxicity in keratinocytes during GAS infection. HaCaT human keratinocytes were infected with GAS at an MOI of 10 for 6 h, and cytotoxicity was determined by ethidium homodimer assay. IL-1β-specific neutralizing antibodies or isotype control antibodies were applied to cells immediately prior to infection. **(A)** Neutralizing antibodies specific for IL-1β significantly reduced cytotoxicity; **(B)** no changes in cytotoxicity were observed in response to non-specific isotype control antibodies. The average of 6 biological replicates is represented for each condition, with error bars representing standard deviation. The overall *p*-values were determined by ANOVA (**A**, *p* = 0.001; **B**, *p* = 0.6944). Dunnett's tests were performed to compare ach WT infection condition to WT+PBS and each uninfected condition to CON+PBS.

Our previous studies have demonstrated that inhibition of p38 MAPK and NFκB significantly reduce SLS-dependent cytotoxicity in keratinocytes during GAS infection (Flaherty et al., 2015). To determine whether the observed SLS-mediated production of IL-1β contributes to cytotoxicity, we treated HaCaT cells with a neutralizing antibody specific for the mature form of IL-1β or an isotype control antibody prior to wild-type GAS infection (Figure S1). Neutralization of IL-1β resulted in a modest, but significant, decrease in GAS-mediated cytotoxicity, indicating that this cytokine contributes to cell death in keratinocytes (Figure 4). Similarly, exogenous treatment of keratinocytes with recombinant IL-1β during wild-type GAS infection resulted in a 5–10% increase in cytotoxicity compared to wild-type infection alone at 6 h post-infection (Figure S2). However, as IL-1β neutralization only partially reduces GAS-mediated cell death in keratinocytes, there are likely to be additional factors downstream of the p38/NFκB cascade that aid in the induction of programmed cell death in keratinocytes during GAS infection.

### Streptolysin S induces IL-1β production during subcutaneous infection of GAS-infected mice

**Figure 5:**
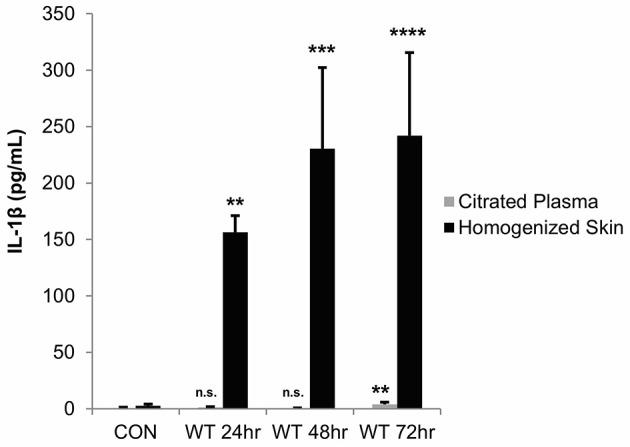
IL-1β production is localized to the skin following subcutaneous GAS infection of mice. Mice were injected subcutaneously in the flank with 10^7^ CFUs of wild-type GAS. Mice were sacrificed at 24, 48, and 72 h, and plasma and skin tissue at the infection site were collected for analysis. IL-1β levels were determined by ELISA. Results from 4 mice were averaged for each condition, and error bars represent standard deviation. The overall *p*-value was determined by ANOVA (citrated plasma, *p* = 0.0019; homogenized skin, *p* < 0.0001). Dunnett's tests were performed to compare each condition to the corresponding uninfected control condition.

As these *in vitro* studies indicated that Streptolysin S is a major contributor to IL-1β production during GAS infection of keratinocytes, we next sought to determine the physiological impacts of IL-1β production in an *in vivo* infection model. We hypothesized that IL-1β production would have a more pronounced effect *in vivo*, as it has been shown to have key roles in the recruitment and activation of cells involved in the innate and adaptive immune responses (Dinarello, 1996; Sahoo et al., 2011; Dinarello et al., 2012). To address this hypothesis, we performed subcutaneous infection of transgenic mice with wild-type GAS and assessed IL-1β production in the skin tissue and plasma at 24, 48, and 72 h post-infection (Figure 5). This infection system was selected based on previous studies by our lab and others, which have demonstrated that SLS drives the formation of necrotic lesions during subcutaneous skin infection in mice (Datta et al., 2005; Mitchell et al., 2009; Higashi et al., 2016). Results from our analyses indicated that IL-1β production is significantly enhanced in the skin at the site of GAS infection, and that it is readily detectable by 24 h post-infection, with levels increasing through 72 h post-infection (Figure 5). At the time points tested, IL-1β was virtually undetectable in the plasma of the infected mice (Figure 5).

**Figure 6:**
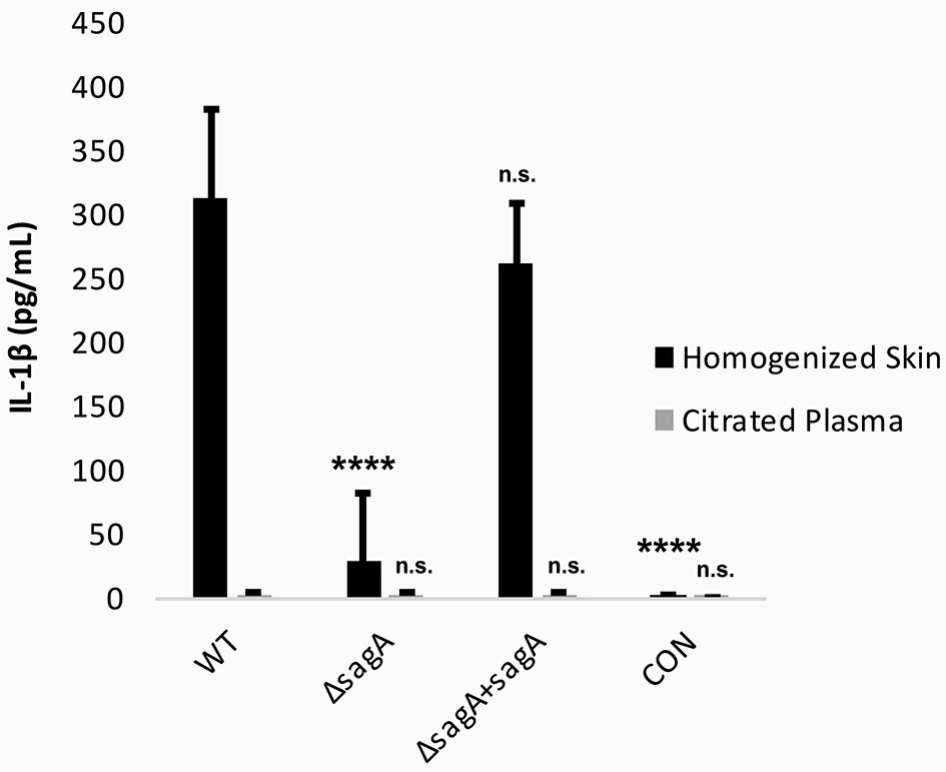
IL-1β is produced in an SLS-dependent manner during GAS subcutaneous skin infection in mice. Mice were injected subcutaneously in the flank with 10^7^ CFUs of GAS. Mice were sacrificed at 72 h, and skin tissue at the infection site as well as plasma were collected for analysis of IL-1β by ELISA. IL-1β protein levels were averaged from 4 mice for each condition. Error bars represent standard deviation. The overall *p*-value was determined by ANOVA (*p* < 0.0001 for homogenized skin; *p* = 0.8712 for citrated plasma). Dunnett's tests were performed to compare each condition to wild-type infection for the corresponding sample type.

To determine whether the observed IL-1β response was SLS-dependent, mice were infected with wild-type, Δ*sagA* and *sagA* complemented GAS. At 72 h post-infection, the mice were sacrificed and plasma samples as well as the skin tissue surrounding the infection site were harvested for IL-1β analysis. We observed significantly enhanced levels of IL-1β in the tissue of mice infected with SLS-containing strains, with much lower IL-1β protein levels in mice infected with the SLS-deficient mutant (Figure 6). IL-1β levels in the plasma of infected mice were not elevated above uninfected controls (Figure 6). GAS-mediated wound formation in the infected mice was consistent with previous reports by our lab and others (Figure S3) and supported the correlation between the presence of SLS and enhanced IL-1β production (Datta et al., 2005; Mitchell et al., 2009; Higashi et al., 2016).

**Figure 7:**
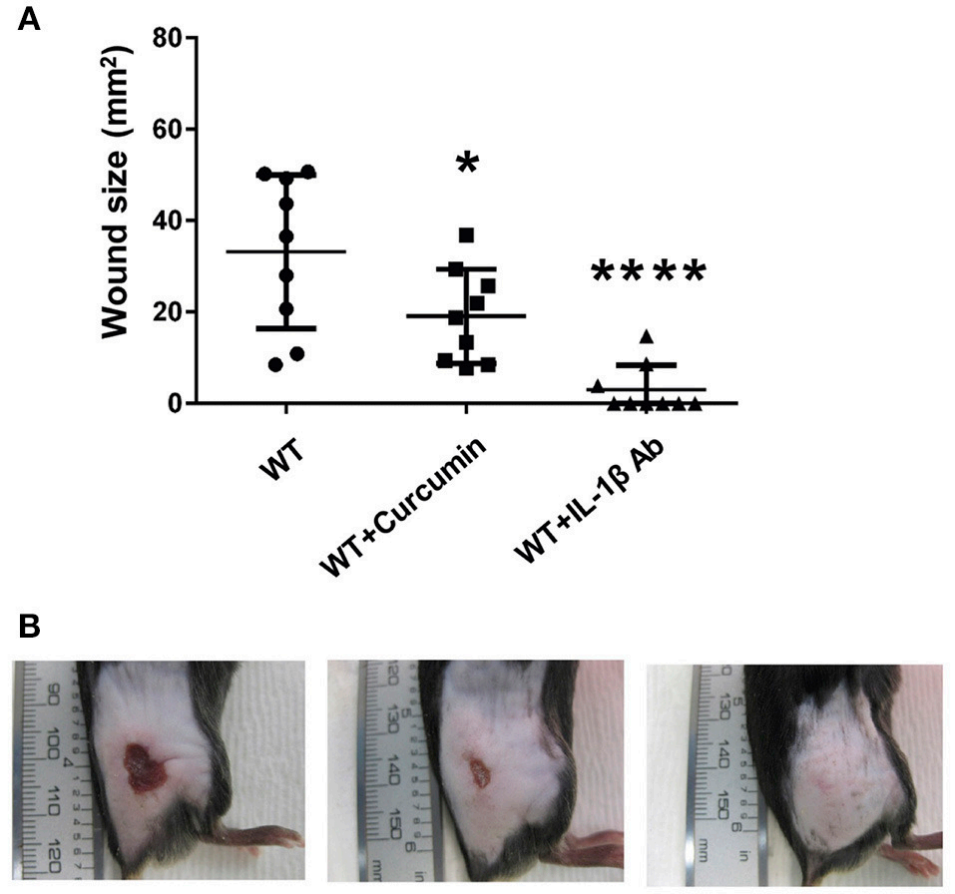
Coadministration of Curcumin and IL-1β neutralizing antibodies with wtGAS reduces SLS-mediated wound formation during subcutaneous GAS infection. Mice were injected subcutaneously in the flank with 10^7^ CFUs of GAS along with Curcumin and IL-1β. Mice were observed for 24 h, at which point the formation of necrotic ulcers was determined. Wound sizes for each treatment condition are shown for each time point. Data were pooled from 3 independent experiments, each containing 3 mice per condition. Error bars represent standard deviation. The overall *p*-values were determined by ANOVA **(A)** *p* < 0.0001. Dunnett's tests were performed to compare each condition to wild-type infection at the corresponding time point. **(B)** Wounds from one representative mouse are shown for each of the treatment conditions shown at 24 h p.i.; from left to right: WT GAS, WT GAS with curcumin, and WT GAS with IL-1β neutralizing antibodies.

Having established that IL-1β production is enhanced during GAS infection in an SLS-dependent manner, we next wanted to determine whether the production or activity of this cytokine could be influenced by inhibitor delivery during infection. To address this question, we injected mice intraperitoneally with the p38 inhibitor SB203580, the NFκB inhibitor curcumin, or a neutralizing antibody specific for IL-1β. Mice were treated with SB203580 or curcumin 48 h prior to infecting them with wild-type GAS, as well as at 0, 24, and 48 h post-infection. Mice receiving the neutralizing antibody were treated at 24 and 48 h post-infection. Lesion formation was monitored at 24, 48, and 72 h post-infection, and at 72 h post-infection, the mice were sacrificed and plasma samples as well as the skin tissue surrounding the infection site were harvested for IL-1β analysis. Our results indicated that in general, inhibition of IL-1β production with intraperitoneal delivery of curcumin, the p38 inhibitor, SB203580, or with the IL-1β neutralizing antibody did not produce robust differences in the average wound sizes (Flaherty, data not shown). Quantification of IL-1β production in the skin of the infected mice revealed that by 72 h post-infection, none of the treatments delivered intraperitoneally were effective at reducing IL-1β levels compared to infected mice treated with only vehicle controls (Flaherty, data not shown). Because intraperitoneal injection of the IL-1β targeting therapies produced minimal changes in GAS-induced tissue damage, we hypothesized that greater efficacy might be achieved by administering these treatments directly at the infection site. To address this, we utilized a co-injection strategy in which each IL-1β targeting therapy was administered subcutaneously along with wild-type GAS. Our results indicated that co-injection of the treatments with GAS significantly reduced average lesion size at 24 h post-infection compared to vehicle controls in the case of both curcumin and IL-1β neutralizing antibodies, with a dramatic reduction being observed in the case of the neutralizing antibodies (Figure 7, Table S2). Treatment with a control isotype antibody did not result in reduction of wound size in infected mice, demonstrating that the neutralizing activity of the antibody treatment is IL-1β specific (Figure S4). Treatment with SB203580 was highly variable, reducing lesion size in some of the infected mice, while greatly exacerbating wound formation in others (Flaherty, data not shown).

**Figure 8:**
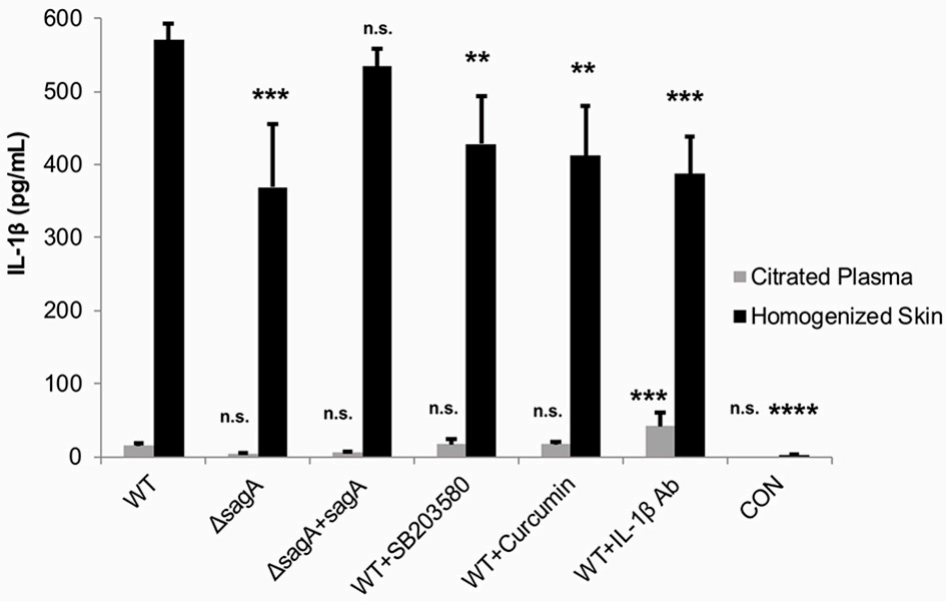
IL-1β targeting therapies reduce mature cytokine levels during subcutaneous GAS infection. Mice were injected subcutaneously in the flank with 10^7^ CFUs of GAS. Mice were sacrificed at 24 h, and skin tissue at the infection site as well as plasma were collected for analysis of IL-1β by ELISA. IL-1β protein levels were averaged from 4 mice for each condition. Error bars represent standard deviation. The overall *p*-value was determined by ANOVA (*p* < 0.0001 for homogenized skin; *p* < 0.0001 for citrated plasma). Dunnett's tests were performed to compare each condition to wild-type infection for the corresponding sample type.

To address whether these treatments were able to significantly alter IL-1β levels at the wound site, ELISA was used to determine cytokine levels in the infected mouse tissue and plasma. Our results indicated that all of the IL-1β targeting treatments did significantly reduce cytokine levels in the tissue, with the most significant reduction occurring in response to administration of the IL-1β neutralizing antibodies (Figure 8). SB203580 and curcumin were less effective at reducing cytokine levels, which is consistent with their reduced capacity to inhibit lesion formation during infection (Figure 8).

We next wanted to determine whether any of the IL-1β targeting therapies were affecting bacterial numbers at the infection site, as inhibition of wound formation could be due to changes in the recruitment and activation of immune cells, a decrease in cytokine-driven host cytotoxicity in the infected tissue, and/or direct bacterial cytotoxicity. To address this question, we again co-injected each of the therapies with wild-type GAS and assessed wound formation at 24 h post-infection. Tissues were then collected to determine CFUs in the skin at the wound site. Our results indicated that treatment with SB203580 or curcumin led to a modest reduction in bacterial numbers at the wound site compared to wild-type-infected mice treated with vehicle controls (Figure S5). Treatment with IL-1β neutralizing antibodies led to a much more significant reduction in CFUs at the wound site, with a loss of approximately one log compared to vehicle controls (Figure S5). Because none of these treatments dramatically reduced bacterial numbers, we do not anticipate that bactericidal activity is the primary cause of reduced wound formation in the presence of these therapies. Further experimentation will be required to uncover the precise mechanism leading to reduced tissue destruction in the presence of IL-1β neutralizing therapies during GAS skin infection.

## Discussion

Our studies demonstrate that Group A *Streptococcal* infection of epithelial keratinocytes modulates the production of inflammatory cytokines. Furthermore, several of these infection-mediated changes in keratinocyte cytokine production are dependent on the secreted toxin Streptolysin S. Our cytokine array analysis and subsequent studies, both in human keratinocytes and in the skin of infected mice, reveal that IL-1β production is significantly enhanced in an SLS-dependent manner during GAS infection. We utilized an immortalized keratinocyte host cell system for our infection studies to maintain consistency and precison, and have utilized this system previously to determine signaling responses due to GAS and SLS (Flaherty et al., 2015). We note the concern that immortalized cell studies have limitations compared to the use of primary keratinocyte cells that may affect the outcome of our cytokine studies. However, overall, the data obtained with our immortalized cells studies *in vitro* were consistent with the *in vivo* mouse studies performed in our report. The results from our *in vitro* studies indicate that SLS-mediated activation of p38 MAPK and NFκB signaling drive the production of this pro-inflammatory response, which contributes to cytotoxicity in infected keratinocytes. Our *in vitro* and *in vivo* studies provide evidence that therapeutic targeting of this signaling cascade, both with small molecule inhibitors and neutralizing antibodies, is a potentially useful strategy to reduce SLS-mediated cell death and tissue destruction during the early stages of invasive GAS skin infection.

Effective control of this potent inflammatory response early in the infection process is likely to be critical not only to control immediate tissue destruction, but also to prevent progression to bacterial dissemination and sepsis. IL-1β has been shown to play an important role in the recruitment of neutrophils and other inflammatory cells to sites of infection, which, in most cases, is beneficial to the host for the containment of microbial infections (Schultz et al., 2002; Mariathasan et al., 2005; Miller et al., 2007; Sahoo et al., 2011). However, IL-1β can also contribute to substantial tissue damage, an overabundance of inflammatory cell recruitment, and uncontrolled cytokine signaling in severe cases, such as during *E. coli*-induced sepsis and *P. aeruginosa-*induced pneumonia (Dinarello, 1996; Schultz et al., 2002, 2003; Lungstras-Bufler et al., 2004; Le Goffic et al., 2006; Zemans et al., 2009; Nathan and Ding, 2010; Sahoo et al., 2011; Vanden Berghe et al., 2014; Kim et al., 2015; Lee et al., 2015; Spohn et al., 2016). In these instances, therapeutic intervention strategies designed to down-regulate IL-1β activity and reduce the activation of its upstream mediators may be particularly important.

The observed changes in bacterial numbers in the skin tissue at 24 h post-infection raise interesting questions about the mechanism at play in our studies and highlight the importance of an appropriate balance of IL-1β signaling during GAS infection. It is possible that in our studies, treatment with IL-1β neutralizing therapies was able to reduce inflammatory signaling to a level that allowed appropriate recruitment of leukocytes to control bacterial spread, while also preventing the initiation of cytokine-driven programmed necrosis and excessive inflammatory cell recruitment that would have otherwise resulted in significant tissue destruction. Tissue destruction resulting both from necrotic signaling cascades as well as from robust immune responses presumably enhances the release of usable nutrients from the damaged tissue, facilitating the growth and dissemination of GAS. These findings are in line with clinical reports demonstrating that increased levels of IL-1β correlate with more severe prognoses in GAS-infected patients presenting with necrotizing fasciitis and/or sepsis (Lungstras-Bufler et al., 2004; Lu et al., 2013).

The ability of IL-1β neutralizing antibodies to dramatically reduce lesion formation and significantly lower IL-1β levels when administered subcutaneously at the infection site is encouraging. We anticipate that effective control of GAS-mediated wound formation may require increased dosage of the treatments tested as the infection progresses, optimization of the vehicle to enhance drug solubility (both for SB203580 and curcumin), and localized treatment administration strategies to ensure the treatments are able to reach the infected site, particularly in cases where tissue destruction has already begun to occur.

The p38 inhibitor, SB203580, has been administered to mice orally at concentrations ranging from 50 to 60 mg/kg daily or intraperitoneally at concentrations ranging from 1 to 1000 mg/kg daily for the treatment of a variety of inflammatory conditions such as *E. coli-*induced sepsis, Dengue virus infection, pneumococcal pneumonia, tuberculosis, arthritis and endometriosis (van den Blink et al., 2001; Inoue et al., 2006; Zhou et al., 2010; Fu et al., 2014). This high degree of variability in reported dosage makes it challenging to predict an appropriate treatment regimen for a rapidly progressing infection like that induced by GAS. As we utilized a fairly modest treatment dose in the studies described here, experimentation with higher doses may be beneficial. An additional complication that likely contributes to SB203580's variable efficacy in our *in vivo* infection system is its poor water solubility (Falck et al., 2013). Although few studies have been conducted to enhance SB203580's solubility for therapeutic use in animal models of disease, such approaches may be worth investigating in the future (Falck et al., 2013). In addition to optimizing drug solubility and dosage, continued efforts to utilize localized treatment approaches to treat subcutaneous GAS infection may be worthwhile. In a recent study, SB203580 and another p38 inhibitor, SB202190, were administered to neonatal mice intradermally to prevent the formation of blisters induced by an autoimmune condition known as pemphigus vulgaris (Berkowitz et al., 2006). It is possible that with an optimized vehicle and a suitable dosage, a similar approach could enhance SB203580's efficacy during GAS skin infection as well.

Like SB203580, curcumin is generally administered either intraperitoneally (50–100 mg/kg per dose) or orally (25–50 mg/kg) for the treatment of a variety of inflammatory diseases such as viral-induced respiratory distress syndrome, *Helicobacter pylori* infection, and sepsis (Vachharajani et al., 2010; Kundu et al., 2011; Avasarala et al., 2013). However, curcumin is notorious for its poor water solubility, which makes its clinical efficacy quite limited at present (Friedrich et al., 2015). Fortunately, recent efforts such as the development of lipid-core nanocapsules (LNC) to serve as drug carrier systems have yielded promising results regarding improved penetration of curcumin into the skin as a topical treatment strategy (Friedrich et al., 2015). Additionally, a recent study in which curcumin was applied to neonatal pig skin as a myristic acid microemulsion demonstrated that this drug delivery approach was 12 times more effective than curcumin dissolved in DMSO for the treatment of *Staphylococcus epidermidis*-induced skin infection (Liu and Huang, 2012). Thus, improved drug delivery approaches such as these could significantly enhance the efficacy of curcumin for the treatment of GAS-induced skin lesion formation.

Neutralizing antibody treatments for the alleviation of inflammatory disorders are generally administered intraperitoneally or intravenously daily or on alternating days at doses ranging from 100 μg to as high as 2 mg (Geiger et al., 1993; Calandra et al., 2000; Vanden Berghe et al., 2014). These therapies have been effective for the treatment of conditions such as arthritis, psoriasis, bacterial sepsis, and toxic shock (Geiger et al., 1993; Calandra et al., 2000; Dinarello et al., 2012; Vanden Berghe et al., 2014). The clinically approved IL-1β neutralizing antibody canakinumab has been found to be highly effective in alleviating the symptoms of a number of chronic inflammatory diseases in humans (Dinarello et al., 2012). However, direct targeting of mature IL-1β with neutralizing antibodies is not the only available therapeutic option to block the activity of this cytokine once it is produced. The clinically approved drug anakinra, which is a recombinant form of the naturally occurring IL-1 receptor antagonist (IL-1Ra), has been shown to effectively reduce inflammation in response to endotoxin-induced sepsis, arthritis, and a number of other inflammatory diseases (Dinarello et al., 2012; Vanden Berghe et al., 2014). Additionally, a chimeric compound (EPI-005) comprised of IL-1Ra and IL-1β was recently developed by Eleven Biotherapeutics, and has already been shown to bind the IL-1 receptor with increased duration and higher affinity than anakinra in pre-clinical trials (Dinarello et al., 2012). Antibodies specific for the IL-1 receptor, rather than IL-1β itself, have also been utilized for the treatment of numerous inflammatory diseases (Cohen et al., 2011; Dinarello et al., 2012). Interestingly, both anakinra and EPI-005 can be applied topically, which might make these therapeutics more ideal for the treatment of GAS skin infection than antibody-based therapies (Dinarello et al., 2012; Okanobo et al., 2012).

The success of our subcutaneous injection treatment strategy to reduce the destructive inflammatory cascades induced by Streptolysin S in the early stages of GAS infection suggests that localized delivery of such therapeutics may be a much more effective means of treating severe GAS skin infections than systemic treatment approaches. We speculate that once tissue destruction begins to occur, treatment strategies that rely on intact vasculature to deliver the therapies to the infection site may be less effective. Other GAS virulence factors have been previously identified as important contributors to IL-1β production during GAS infection (Hackett and Stevens, 1992; LaRock et al., 2016; Valderrama et al., 2017). SpeA and SLO have been shown to trigger potent interleukin-1 production in human monocyte cell lines, and soluble forms of M1 protein induced caspase-1-dependent NLRP3 inflammasome activation, resulting in the maturation and release of IL-1β (Valderrama et al., 2017). These studies suggest that IL-1β is likely to be a key driver of GAS pathogenesis; however, it is possible that diverse infection model systems as well as strain-specific differences in virulence factor structure and function may be responsible for some of the differences in the mechanistic details of IL-1β production. Timmer et al. demonstrated the rapid apoptosis of macrophages and neutrophils upon internalization of GAS (Timmer et al., 2009). In this model, internalized GAS required SLO for promoting cellular apoptosis, but not other factors such as SpeB, cell-anchored M1 protein or SLS. These findings support the idea that even though GAS-mediated activation of IL-1β is likely to vary depending on infection context and bacterial strain, precise therapeutic targeting of IL-1β during the many stages of GAS infection may be an attractive strategy regardless of specific strain or infection type. Furthermore, it highlights how different types of GAS infections might require different modes of IL-1β therapeutic delivery, depending on the disease state and virulence factors involved. In our future studies to mitigate the effects of SLS-driven inflammatory signaling during subcutaneous GAS skin infection, we will seek to incorporate some of the recent advances pertaining to treatment dosage and delivery, particularly for our small molecule inhibitors, as well as examining alternative IL-1β blockade mechanisms. We anticipate that these studies will aid in the development of new therapeutic strategies to minimize the devastating effects of severe Group A *Streptococcal* disease.

## Author contributions

RF, DD, VP, FC, and SL: designed the overall project and experimental aims; RF, DD, KC, JR: performed experimental work and analyzed the results; RF and SL: wrote the paper, with section contributions from DD, VP, and FC. All authors contributed to the proofreading and editing of the paper.

### Conflict of interest statement

The authors declare that the research was conducted in the absence of any commercial or financial relationships that could be construed as a potential conflict of interest.

## Supplementary material

Figure S1 IL-1β production during GAS infection can be significantly reduced through the use of an IL-1β-specific neutralizing antibody. HaCaT human keratinocytes were infected with GAS at an MOI of 10 for 6 h, and cell culture media was collected for cytokine analysis. IL-1β-specific neutralizing antibodies or isotype control antibodies were applied to cells 1 h prior to infection. Following infection, the cell culture media was incubated for 16 h with protein A/G beads to bind antibody-IL-1β complexes. The samples were then centrifuged and the remaining IL-1β was determined by ELISA. The average of three biological replicates, each with 3 technical replicates, are represented for each condition, with error bars representing standard deviation. The overall *p*-value was determined by ANOVA (**A**, *p* < 0.0001; **B**, *p* = 0.226). Dunnett's tests were performed to compare each WT infection condition to WT+PBS and each uninfected condition to CON+PBS.

Figure S2 Exogenous IL-1β enhances GAS-mediated cytotoxicity in keratinocytes. HaCaT human keratinocytes were infected with GAS at an MOI of 10 for 6 h, and cytotoxicity was determined by ethidium homodimer assay. Exogenous IL-1β was applied to keratinocytes 1 h prior to infection with wild-type GAS. The average of 4 biological replicates is represented for each condition, with error bars representing standard deviation. The overall *p*-value was determined by ANOVA (*p* < 0.0001), and Dunnett's tests were performed to compare each WT infection condition to WT+VC and each uninfected condition to CON+VC. VC refers to vehicle control.

Figure S3 Necrotic wound size is highly dependent on SLS production by GAS. Mice were injected subcutaneously in the flank with 10^7^ CFUs of GAS. Mice were observed for 72 h, and the formation of necrotic ulcers was monitored daily. Mice were sacrificed at 72 h, and skin tissue at the infection site as well as plasma were collected for analysis. Wound sizes for each treatment condition, averaged from 4 mice, are shown for each time point **(A)** 24 h; **(B)** 48 h; and **(C)** 72 h. Error bars represent standard deviation. The overall *p*-values were determined by ANOVA (24 h, *p* = 0.0011; 48 h, *p* = 0.0042; 72 h, *p* = 0.0015). Dunnett's tests were performed to compare each condition to wild-type infection at the corresponding time point. **(D)** Wounds from one representative mouse are shown for each of the conditions tested at 72 h p.i.; from left to right: WT GAS, ΔsagA GAS, ΔsagA+sagA GAS.

Figure S4 Coadministration of IL-1β Isotype control antibody does not result in decrease in wound formation during subcutaneous GAS infection. Mice were injected subcutaneously in the flank with 10^7^ CFUs of GAS along with isotype control antibody at the site of injection or vehicle control. Mice were observed for 24, 48, and 72 h, at which point the formation of necrotic ulcers was determined. **(A)** Wound sizes for each treatment condition are shown for each time point. Data represent six mice used per condition. Error bars represent standard deviation. The overall *p*-values were determined by ANOVA **(A)** p < 0.0001. Dunnett's tests were performed to compare each condition to wild-type infection at the corresponding time point. **(B)** Wounds from one representative mouse are shown for each of the treatment conditions shown at 24 h p.i.; from left to right: WT GAS, WT GAS with isotype control antibodies. **(C)** CFU Determination. Mice were sacrificed at 72 h, and skin tissue at the infection site was collected and homogenized immediately. Samples from each mouse were serially diluted and plated in quadruplicate to determine CFUs. Data were pooled from two independent experiments, each containing 3 mice per condition. The average CFU counts are shown, with error bars representing standard deviation. The overall *p*-value was determined by ANOVA (*p* < 0.0001).

Figure S5 IL-1β targeting therapies have minimal effect on colony forming units (CFUs) recovered from the infection site at 24 h post-infection. Mice were injected subcutaneously in the flank with 10^7^ CFUs of GAS. Mice were sacrificed at 24 h, and skin tissue at the infection site was collected and homogenized immediately. Samples from each mouse were serially diluted and plated in quadruplicate to determine CFUs. Data were pooled from two independent experiments, each containing 3 mice per condition. The average CFU counts are shown, with error bars representing standard deviation. The overall *p*-value was determined by ANOVA (*p* < 0.0001). Dunnett's tests were performed to compare each condition to wild-type infection.

Table S1 Annotations for Significantly Altered Cytokines in GAS Infection.

Table S2 Wound Sizes Following Subcutaneous GAS Infection in Mice with Treatments Administered Subcutaneously. Mice were injected subcutaneously in the flank with 10^7^ CFUs of GAS along with treatments administered subcutaneously. Mice were observed for 24 h, at which point the formation of necrotic ulcers was determined. Wound sizes for each treatment condition are shown for each time condition. Data were pooled from 3 independent experiments, each containing 3 mice per condition. Individual wound sizes with highest outlying values are indicated in red. Wounds are measured for each of the treatment conditions tested: WT GAS, Δ*sagA* GAS, Δ*sagA*+*sagA* GAS, WT GAS with SB203580, WT GAS with curcumin, and WT GAS with IL-1β neutralizing antibodies.
