# Supplementary material for: Neutralization of Streptolysin S-Dependent and Independent Inflammatory Cytokine IL-1β Activity Reduces Pathology During Early Group A Streptococcal Skin Infection
Source: Front Cell Infect Microbiol. 2018 Jul 3;8:211. doi: 10.3389/fcimb.2018.00211 (PMC6037840; doi:10.3389/fcimb.2018.00211)
Supplement: Figure S1 — IL-1β production during GAS infection can be significantly reduced through the use of an IL-1β-specific neutralizing antibody. HaCaT human keratinocytes were infected with GAS at an MOI of 10 for 6 h, and cell culture media was collected for cytokine analysis. IL-1β-specific neutralizing antibodies or isotype control antibodies were applied to cells 1 h prior to infection. Following infection, the cell culture media was incubated for 16 h with protein A/G beads to bind antibody-IL-1β complexes. The samples were then centrifuged and the remaining IL-1β was determined by ELISA. The average of three biological replicates, each with 3 technical replicates, are represented for each condition, with error bars representing standard deviation. The overall p-value was determined by ANOVA (A, p < 0.0001; B, p = 0.226). Dunnett's tests were performed to compare each WT infection condition to WT+PBS and each uninfected condition to CON+PBS. [file Data_Sheet_1.PDF]

Table S1: Annotations for Significantly Altered Cytokines in GAS Infection

Cytokine	Function (Uniprot.org)
**GM-CSF**	Stimulates the growth and differentiation of granulocytes, macrophages, eosinophils and erythrocytes.
**IL-1β**	Potent proinflammatory cytokine which was initially discovered as the major endogenous pyrogen. Induces prostaglandin synthesis, neutrophil influx and activation, T-cell activation and cytokine production, B-cell activation and antibody production, and fibroblast proliferation and collagen production. Promotes Th17 differentiation of T-cells.
**IL-6**	Potent inducer of the acute phase response. Plays an essential role in the final differentiation of B-cells into Ig-secreting cells. Involved in lymphocyte and monocyte differentiation. Acts on B-cells, T-cells, hepatocytes, hematopoietic progenitor cells and cells of the CNS. Required for the generation of Th17 cells.
**MIF**	Pro-inflammatory cytokine. Involved in the innate immune response to bacterial pathogens. The expression of MIF at sites of inflammation suggests a role as mediator in regulating the function of macrophages in host defense. Counteracts the anti-inflammatory activity of glucocorticoids.
**MDC (CCL22)**	May play a role in the trafficking of activated/effector T-lymphocytes to inflammatory sites and other aspects of activated T-lymphocyte physiology. Chemotactic for monocytes, dendritic cells and natural killer cells. Mild chemoattractant for primary activated T-lymphocytes and a potent chemoattractant for chronically activated T-lymphocytes but has no chemoattractant activity for neutrophils, eosinophils, and resting T-lymphocytes. Binds to CCR4.
**MIP-1δ**	Chemotactic factor that attracts T-cells and monocytes, but not neutrophils, eosinophils, or B-cells. Acts mainly via CC chemokine receptor CCR1. Also binds to CCR3.
**Osteoprotegerin**	Acts as decoy receptor for TNFSF11/RANKL and thereby neutralizes its function in osteoclastogenesis. Inhibits the activation of osteoclasts and promotes osteoclast apoptosis in vitro. Bone homeostasis seems to depend on the local ratio between TNFSF11 and TNFRSF11B. May also play a role in preventing arterial calcification. May act as decoy receptor for TNFSF10/TRAIL and protect against apoptosis. TNFSF10/TRAIL binding blocks the inhibition of osteoclastogenesis.
**TIMP-2**	Complexes with metalloproteinases (such as collagenases) and irreversibly inactivates them by binding to their catalytic zinc cofactor. Known to act on MMP-1, MMP-2, MMP-3, MMP-7, MMP-8, MMP-9, MMP-10, MMP-13, MMP-14, MMP-15, MMP-16 and MMP-19.
**I-309 (CCL1)**	Cytokine that is chemotactic for monocytes but not for neutrophils. Binds to CCR8.
**IL-1α**	Produced by activated macrophages, IL-1 stimulates thymocyte proliferation by inducing IL-2 release, B-cell maturation and proliferation, and fibroblast growth factor activity. IL-1 proteins are involved in the inflammatory response, being identified as endogenous pyrogens, and are reported to stimulate the release of prostaglandin and collagenase from synovial cells.
**IL-2**	Produced by T-cells in response to antigenic or mitogenic stimulation, this protein is required for T-cell proliferation and other activities crucial to regulation of the immune response. Can stimulate B-cells, monocytes, lymphokine-activated killer cells, natural killer cells, and glioma cells.

Table S1 Continued:

Cytokine	Function (Uniprot.org)
**IL-15**	Stimulates the proliferation of T-lymphocytes; requires interaction with components of IL-2R, including IL-2R beta and probably IL-2R gamma but not IL-2R alpha.
**MCP-3 (CCL7)**	Chemotactic factor that attracts monocytes and eosinophils, but not neutrophils. Augments monocyte anti-tumor activity. Also induces the release of gelatinase B. Binds heparin, CCR1, CCR2 and CCR3.
**MIG (CXCL9)**	Affects the growth, movement, or activation state of cells that participate in immune and inflammatory response. Chemotactic for activated T-cells. Binds to CXCR3.
**MCP-1**	Chemotactic factor that attracts monocytes and basophils but not neutrophils or eosinophils. Augments monocyte anti-tumor activity. Has been implicated in the pathogenesis of diseases characterized by monocytic infiltrates, like psoriasis, rheumatoid arthritis or atherosclerosis. May be involved in the recruitment of monocytes into the arterial wall during the disease process of atherosclerosis.
**MCSF**	Cytokine that plays an essential role in the regulation of survival, proliferation and differentiation of hematopoietic precursor cells, especially mononuclear phagocytes, such as macrophages and monocytes. Promotes the release of proinflammatory chemokines, and thereby plays an important role in innate immunity and in inflammatory processes. Plays an important role in the regulation of osteoclast proliferation and differentiation, bone resorption, and bone development. Promotes reorganization of the actin cytoskeleton, regulates formation of membrane ruffles, cell adhesion and cell migration. Plays a role in lipoprotein clearance.
**MIP-1b (CCL4)**	Inflammatory and chemokinetic properties. Binds to CCR5. One of the major HIV-suppressive factors produced by CD8+ T-cells. MIP-1-beta(3-69) is also a ligand for CCR1 and CCR2 isoform B.
**Thrombopoietin**	Lineage-specific cytokine affecting the proliferation and maturation of megakaryocytes from their committed progenitor cells. It acts at a late stage of megakaryocyte development. It may be the major physiological regulator of circulating platelets.

Table S2: Wound Sizes Following Subcutaneous GAS Infection in Mice with Treatments Administered Subcutaneously

24hrs Post-Infection Wound Sizes (sq. mm)				
	WT	WT+SB203580	WT+Curcumin	WT+IL-1β Ab
	20.68	154.35	36.93	3.92
	50.72	33.33	77.51	36.89
	49.21	139.33	18.74	8.71
	103.47	53.41	29.46	0.00
	43.68	17.54	9.35	0.00
	28.01	27.96	13.41	0.00
	36.54	9.81	7.74	0.00
	8.51	16.09	25.70	14.76
	50.20	86.72	8.44	0.00
	10.88	200.97	21.90	0.00
AVE	40.19	73.95	24.92	6.43
SD	25.91	64.70	19.80	11.21

24hrs Post-Infection Wound Sizes (sq. mm)						
	WT	ΔsagA	ΔsagA+sagA	WT+SB203580	WT+Curcumin	WT+IL-1β Ab
	20.68	0.00	56.55	154.35	36.93	3.92
	50.72	17.96	10.44	33.33	77.51	36.89
	49.21	23.13	45.78	139.33	18.74	8.71
	103.47	25.41	27.07	53.41	29.46	0.00
	43.68	69.23	58.39	17.54	9.35	0.00
	28.01	0.00	0.00	27.96	13.41	0.00
	36.54	0.00	0.00	9.81	7.74	0.00
	8.51	13.19	48.17	16.09	25.70	14.76
	50.20	2.67	20.35	86.72	8.44	0.00
	10.88	66.44	6.32	200.97	21.90	0.00
AVE	40.19	21.80	27.31	73.95	24.92	6.43
SD	25.91	24.77	22.05	64.70	19.80	11.21

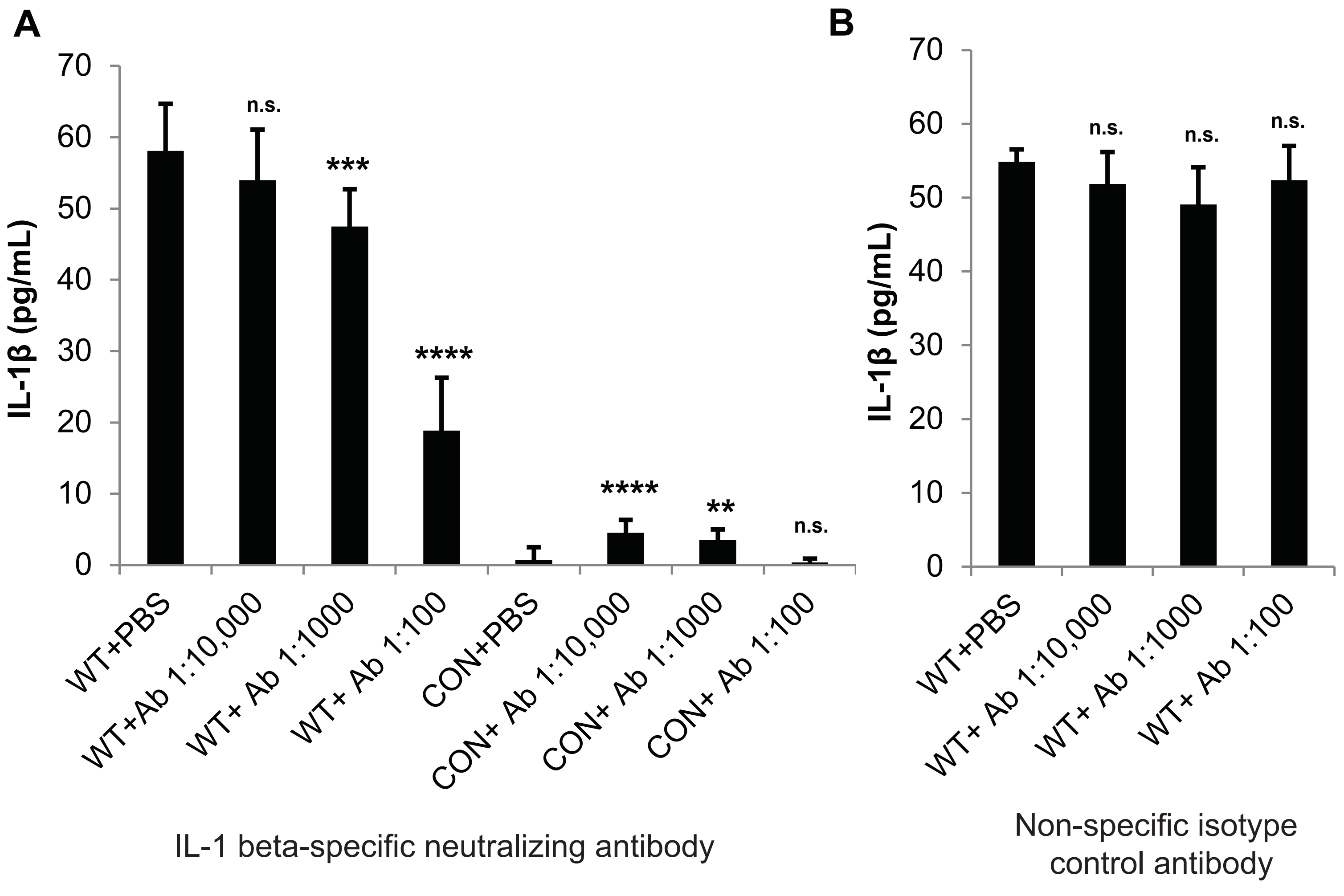

A
B
IL-1β (pg/mL)
70
60
50
40
30
20
10
0
n.s.
***
****
****
**
n.s.
WT+PBS
WT+Ab 1:10,000
WT+ Ab 1:1000
WT+ Ab 1:100
CON+PBS
CON+ Ab 1:10,000
CON+ Ab 1:1000
CON+ Ab 1:100
IL-1 beta-specific neutralizing antibody
IL-1β (pg/mL)
n.s.
n.s.
n.s.
WT+PBS
WT+ Ab 1:10,000
WT+ Ab 1:1000
WT+ Ab 1:100
Non-specific isotype control antibody

Figure S1

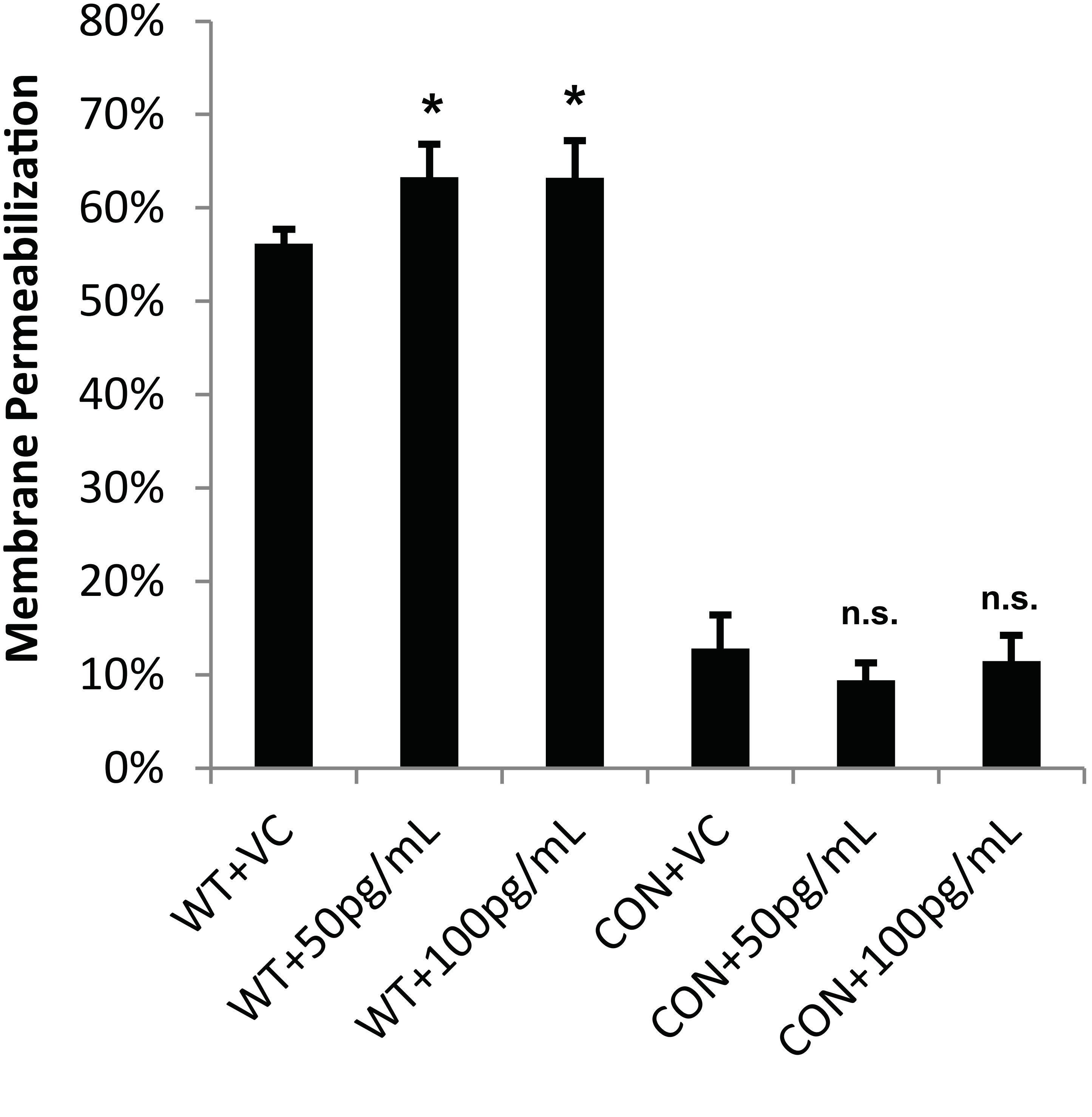
Membrane Permeabilization
80%
70%
60%
50%
40%
30%
20%
10%
0%
*
*
n.s.
n.s.
WT+VC
WT+50pg/mL
WT+100pg/mL
CON+VC
CON+50pg/mL
CON+100pg/mL

Figure S2

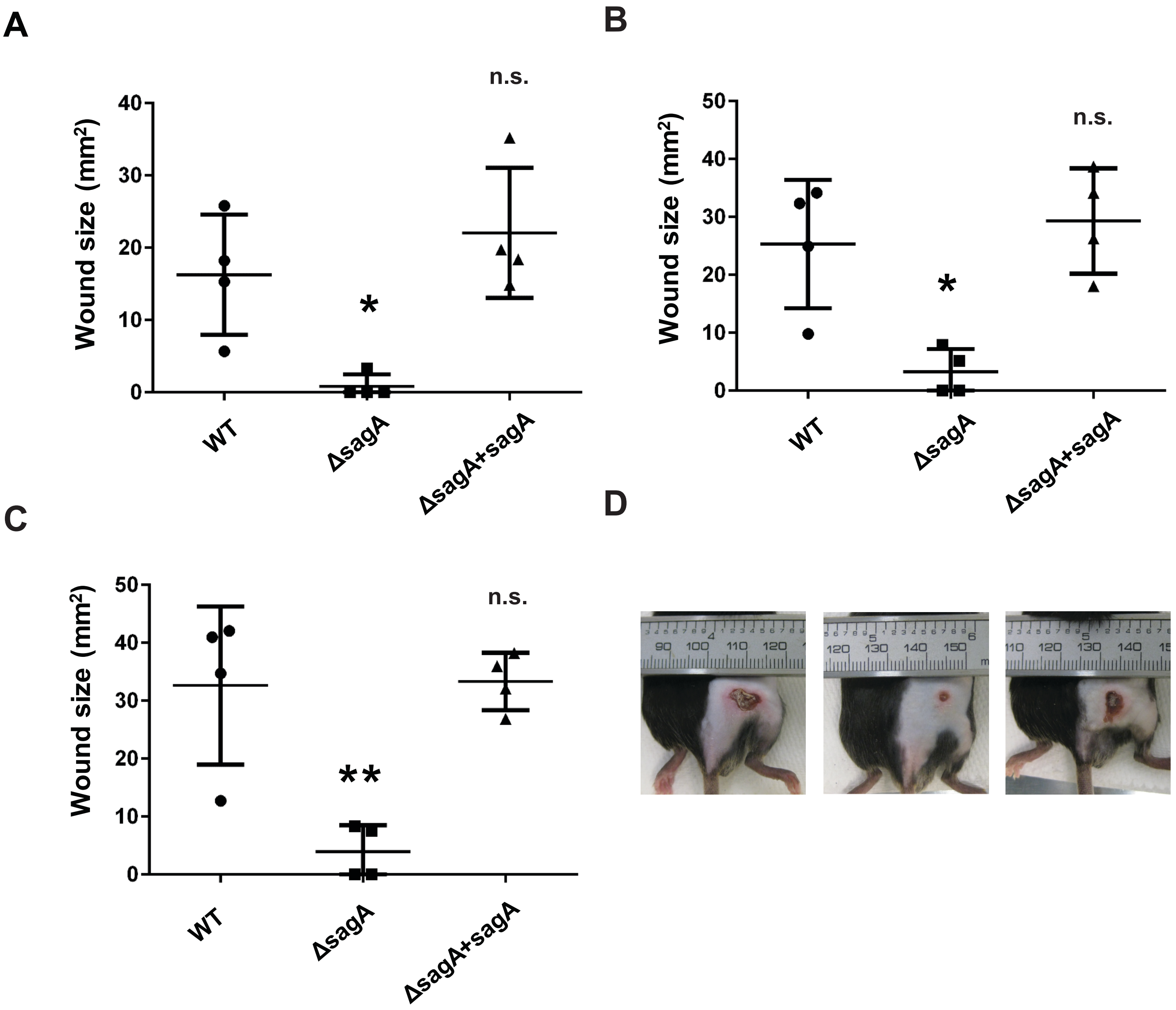
A
B
C
D
Wound size (mm²)
0
10
20
30
40
50
WT
ΔsagA
ΔsagA+sagA
*
**
n.s.
90 100 110 120
120 130 140 150
110 120 130 140

Figure S3

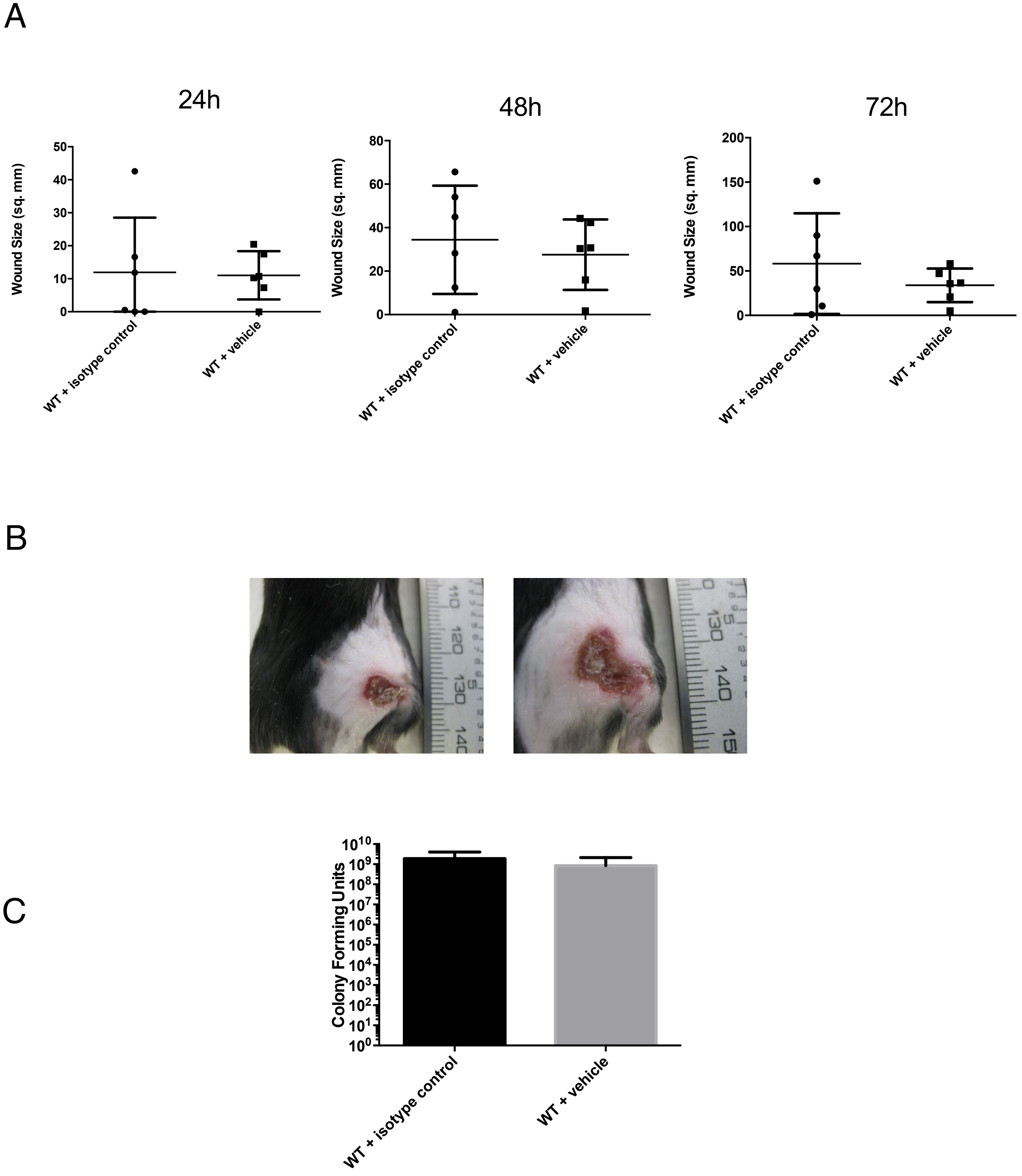
A
24h
48h
72h
Wound Size (sq. mm)
0
10
20
30
40
50
60
80
100
150
200
WT + isotype control
WT + vehicle
B
C
Colony Forming Units
10^0
10^1
10^2
10^3
10^4
10^5
10^6
10^7
10^8
10^9
10^{10}

Figure S4

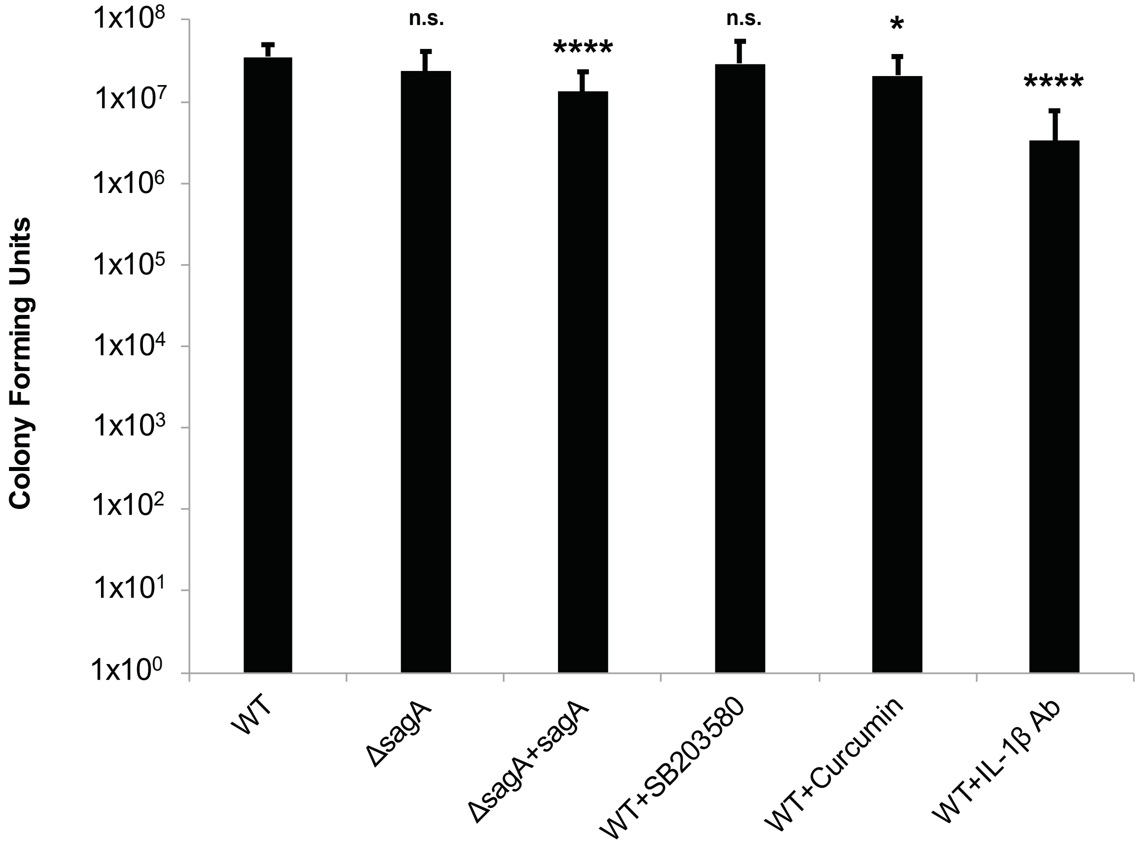

Colony Forming Units
1x10^8
1x10^7
1x10^6
1x10^5
1x10^4
1x10^3
1x10^2
1x10^1
1x10^0
n.s.
****
n.s.
*
****
WT
ΔsagA
ΔsagA+sagA
WT+SB203580
WT+Curcumin
WT+IL-1β Ab

Figure S5
